# Element leaching from green liquor dregs from 16 Swedish pulp and paper mills between 2017 and 2019

**DOI:** 10.1038/s41598-026-51421-1

**Published:** 2026-05-09

**Authors:** Nanna Stahre, Lotta Sartz, Mattias Bäckström

**Affiliations:** 1 Örebro University School of Science and Technology: Orebro universitet Institutionen for naturvetenskap och teknik, Örebro University, Örebro, 701 82 Sweden; 2 Bergskraft Bergslagen AB, Södra Kungsvägen 49, Kumla, 692 30 Sweden

**Keywords:** Green liquor dregs, Mining waste, Co-disposal, Mining site remediation, Acid mine drainage, Acid rock drainage, Environmental sciences, Environmental chemistry, Environmental impact

## Abstract

Several studies have found that green liquor dregs (GLD) can be a possible remediant for remediation of oxidised sulfidic mine sites. GLD is a heterogeneous alkaline waste from the pulp and paper industry, and as such, broad scale utilization requires environmental properties to be characterized in accordance with Swedish law. Among these properties, water soluble leaching properties of GLD must be examined. In this study samples were collected from 16 mills at five different occasions, approximately 6 months apart, totalling 71 samples. Leaching was performed in sequential steps at a L/S ratio 2 and 8 on wet samples corresponding to 25 g d.w, resulting in a total L/S ratio of 10. The liquid phase was analysed for electrical conductivity, pH, alkalinity, anions and element concentrations, after which correlation calculations and geochemical calculations were performed. Leaching of sodium and potassium was as expected very high while leaching of calcium, magnesium and iron was low. Trace elements leach generally less than ≤ 5%. Na, K, As, Ba, Na, K, Rb and U are present mainly as easily soluble salts that are washed out over time while Ag, Co, Cr, Cu, Ni, Pb, Se, Sr, V and Zn are mainly solubility controlled. Hydroxides are indicated by geochemical calculations to be the solubility controlling phases for Cd, Co, Ni, Zn and in some cases also for lead, while oxides are the suggested controlling phase for cobalt. For copper the results suggested both oxides and hydroxides as controlling phases. In general, most elements of concern had low enough leaching rates that their impact will be moderate as leaching in the study was under optimal conditions and in situ application is expected to lower leching rates further. When comparing the chemistry of leachates from GLD with the chemistry of leachates from acidic mining waste there are environmental benefits to be had from co-disposal even if GLD is not inert. This combined with the fact that both short term and long term buffering capacity is excellent indicates that GLD is suitable for remediation of acidic mining waste.

**Supplementary Information:**

The online version contains supplementary material available at 10.1038/s41598-026-51421-1.

## Introduction

Sweden has a long history of mining where the oldest known mines are from the 12th century in the Bergslagen area. While sulfidic mines were the second most common type of mine in Bergslagen after iron oxide mines, they are the main type in the Skellefte ore field. Old closed sulfidic mines, ranging from several centuries to less than 30 years since closure can be found throughout most of Sweden, with only a few counties in the south not having any known sulfidic mines^1^. Almost all of these older mine sites are considered orphaned and very few have been remediated, resulting in an abundance of small sites that are actively leaching effluent with low pH and high elemental concentration. In addition to this, several sites are also marked as cultural heritage sites meaning remediation is restricted to non-appearance changing techniques. Due to both the practical problems of small site remediation as well as the cost of remediation, which falls on either the land owner or the Swedish government, research has been ongoing to find a suitable remediant. While being a large problem in Sweden this phenomenon is not exclusive to Sweden. Similar sites can be found all over the world, for example in the Americas, one can find similar sites in the US, Canada and Chile. In Africa sites can be found in South Africa and in the Zambian Copperbelt among others, and in Asia notable sites include Jinchuan, Baiyin, East Kunlun. This makes the problem of cheap and easy remediation of sulfidic mine sites a global problem.

Different kinds of waste have been examined as using a waste will result in a more circular economy. It will also have the benefit of significant cost reduction, mainly for remediation itself but also due to decreased cost of waste disposal for the waste used as a remediant. A low cost remediant that does not require significant alteration or production has become a deciding factor for remediation in many countries due to the state of both local and global economy. As sulfidic mine sites are acid generators, alkaline wastes are the best suited as co-disposal of sulfidic mining waste. The alkaline waste will create a near neutral pH environment, meaning that leaching of several elements should decrease due to sorption and co-precipitation. This would decrease the environmental impact from effluent with high/low pH and would also result in a combined effluent that has lower environmental impact than that from the wastes on their own^2,3^.

Several researchers^3–7^ among others have examined different types of alkaline wastes and found that green liquor dregs (GLD) is one of the most suitable wastes to be utilized for acid rock drainage (ARD) remediation. GLD is generated during chemical recovery at pulp and paper mills and consists of mainly leftover organic material, non-processed elements from wood pulping, spent cooking chemicals that have not been washed out, and in some cases added lime mud containing calcite. It is available in high quantities as almost all GLD is landfilled and can often be sourced locally as there are mills distributed over most of Sweden. GLD has high pH, low hydraulic conductivity, high buffering capacity and adheres to surfaces when mixed with mining waste, reducing the risk of the material being washed out. These characteristics make GLD suitable for remediation of acid rock drainage and acidic mine waste by means of co-disposal and injection. Injection is preferable compared to traditional co-disposal, by means of mixing or landfill cover, as injection is less invasive and more cost effective^6^. It can also be performed without affecting the site’s appearance and can more easily be performed at inaccessible and/or cultural sites. From a global perspective, most countries have pulp and paper mills and thus generation of GLD in need of disposal, and while the mills are not always located in as close proximity as in Sweden, the transportation is still often shorter than if a remediation product needs to be shipped internationally.

Use of a novel remediation technique usually requires several pilot studies before permission is granted by national and local authorities to use the technique more broadly. For small sites it is not always practical to perform several pilot studies as this may incur higher costs than an actual remediation. It is therefore important for the authorities to be able to grant a wider permit that is not site specific. To be able to issue this kind of large-scale general permits, the material used needs to be characterised on a larger scale. This includes leaching tests, performed either as a percolation test or as a sequential shake test at L/S 2 and L/S 8 where the total leached amount is calculated as L/S 10. This method is a direct implementation of the European waste disposal directive (1999/31/EG) into Swedish law.

A few prior studies have performed leaching tests on GLD^3,8,9^. However, these studies were only based on a few samples. The aim of this study is to investigate the characteristics of GLD leachate from several Swedish mills on several occasions. Further, the study aims to determine the variation in the leachate chemistry of a large sample population and decide if GLD is a suitable remediant for treating acid generating waste rock. While pilot studies and field studies are necessary to fully demonstrate broad regulatory suitability, this study aims to increase the understanding of the general leaching characteristics of GLD, and show the potential suitability of GLD, and aid in determining whether GLD is suitable as a generalised waste remediator, instead of having a single case suitability.

## Method

### Generation of GLD

GLD is generated during chemical recovery of white liquor (cooking chemicals consisting of sodium hydroxide (NaOH) and sodium sulfate (Na_2_SO_4_)), i.e. the kraft cooking process. In rare cases sodium sulfite (Na(HSO_3_)_2_) is used instead of sodium sulfate, i.e. the sulfite cooking process^10,11^. After the pulp is separated out post-cooking, it is called weak black liquor, and consists of spent cooking chemicals, left over wood constituents (i.e. organic material) and water. The weak black liquor is evaporated in order to lower the water content resulting in strong black liquor that is then combusted in order to burn off as much remaining organic matter as possible^7,^^12,13^. The resulting smelt is dissolved using weak liquor (diluted cooking chemical solution). This solution, called green liquor, consists of both a solid and a liquid phase. The solids are mechanically separated, becoming green liquor dregs, while the filtered green liquor is recausticized into new white liquor^7,^^12,13^.

### Sample collection

**Fig. 1:**
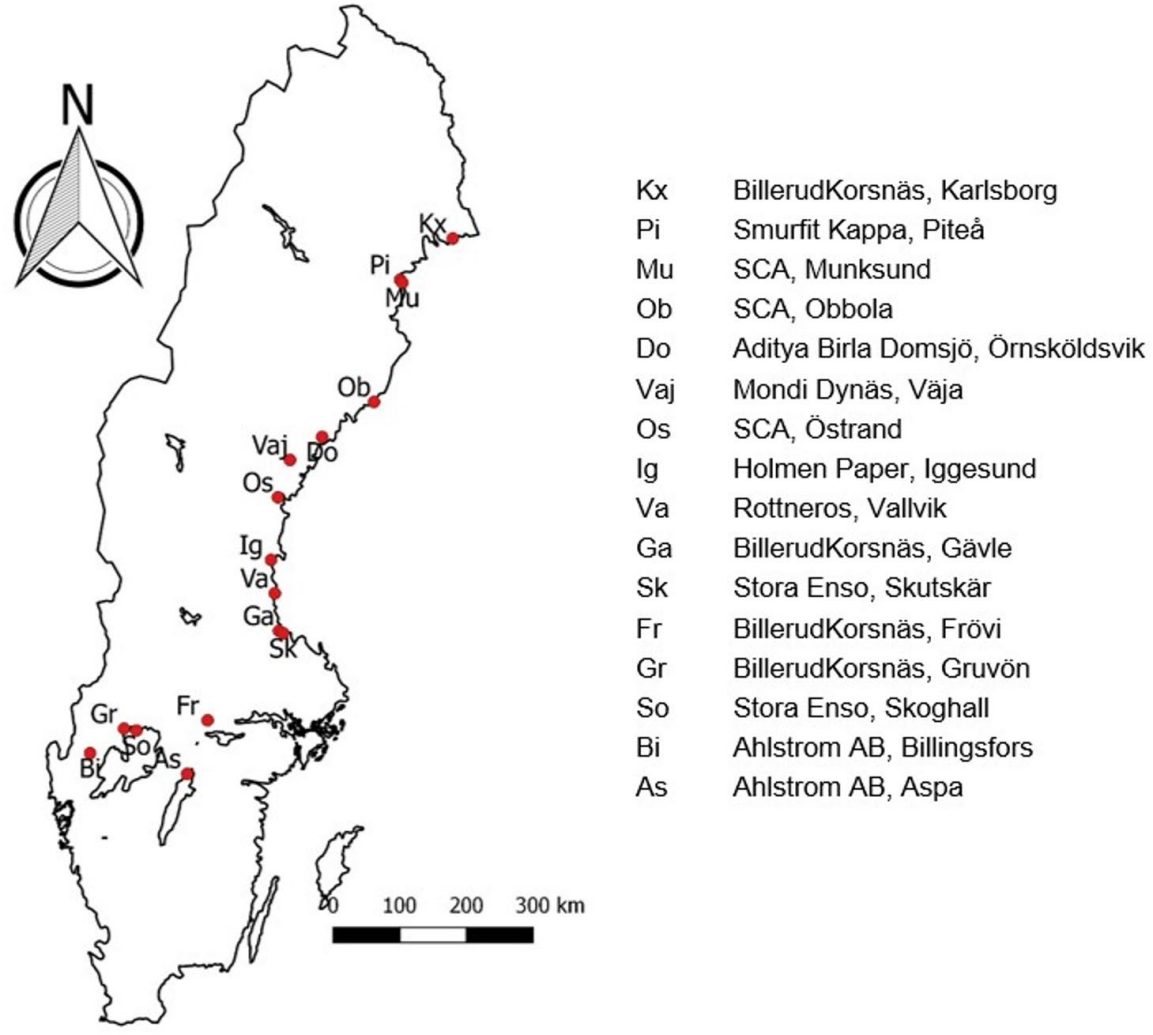
Map showing the locations of the participating mills in Sweden (map was generated by QGIS Desktop 2.14.13, https://www.qgis.org/downloads-list/, with data from Lantmäteriet (Swedish mapping, cadastral and land registration authority) as a basis).

Samples were collected from sixteen Swedish mills in 2017 (spring and fall), 2018 (spring and fall) and in spring 2019. The Domsjö mill is a biorefinery that uses the sodium sulfite cooking process while the Gruvön mill uses both the kraft and the sulfite cooking process. All other mills in the study use only the kraft process^7^. Location of the mills can be seen in Fig. 1.

Sampling was performed by the mill personnel at the end of the mill’s GLD processing line with a sample volume of 4–6 L (wet samples). Not all mills submitted all five samples, resulting in a total of 71 samples. Domsjö, Piteå and Skoghall did not send any sample for spring 2017, but sent samples for the rest of the sample series (Domsjö sent two samples for fall 2017). Väja only sent the spring 2017 and the fall 2018 samples, and Skutskär only sent the spring 2017 sample. All samples were single samples, except for the Billingsfors spring 2017 sample and all of the Östrand samples which were composite samples, Table 1.

**Table 1:** Received GLD samples and sample type, most samples were grab samples while composite samples consisted of three subsamples taken during a 24-hour period.

Mill	Sample series				
	Spring 2017 *S17*	Fall 2017 *F17*	Spring 2018 *S18*	Fall 2018 *F18*	Spring 2019 *S19*
BillerudKorsnäs Karlsborg	Single	Single	Single	Single	Single
Smurfit Kappa Piteå	No sample	Single	Single	Single	Single
SCA Munksund	Single	Single	Single	Single	Single
SCA Obbola	Single	Single	Single	Single	Single
Aditya Birla Domsjö	No sample	2 singles	Single	Single	Single
Mondi Dynäs Väja	Single	No sample	No sample	Single	No sample
SCA Östrand	Composite	Composite	Composite	Composite	Composite
Holmen Paper Iggesund	Single	Single	Single	Single	Single
Rottneros Vallvik	Single	Single	Single	Single	Single
BillerudKorsnäs Gävle	Single	Single	Single	Single	Single
Stora Enso Skutskär	Single	No sample	No sample	No sample	No sample
BillerudKorsnäs Frövi	Single	Single	Single	Single	Single
BillerudKorsnäs Gruvön	Single	Single	Single	Single	Single
Munksjö Paper Billingsfors	Composite	Single	Single	Single	Single
Munksjö Paper Aspa	Single	Single	Single	Single	Single
Stora Enso Skoghall	No sample	Single	Single	Single	Single

The cursive designation in sample series is the shortened sample series name used in figures.

### Leaching experiments

Leaching was performed in two sequential steps, at L/S 2 and L/S 8, giving a total accumulated L/S of 10. This is in accordance with the standard leaching protocol SS-EN 12457-3^14^ for determination of leaching properties of waste that is not regularly produced (as specified by Swedish regulation). This method was chosen over percolation tests, which are generally used for determination of leaching properties of regularly produced waste, due to economical and practical constraints.

For the first leaching step (L/S 2) wet (as is from the mill) samples corresponding to 25 g d.w. were placed in 250 ml centrifuge bottles to which 50 ml of ultrapure water (18.2 MΩ resistance) was added. Wet sample weight was calculated using data from Stahre et al.,^7^, Table 1 in Supplementary data. The samples were shaken in an end-over-end tumbler for 24 h ± 30 min. After shaking, all samples except those from the Aspa mill were centrifuged at 20 000 G for 20 min, after which the liquid phase was poured off and saved for further analysis. The Aspa mill samples were first centrifuged for 20 min at 20 000 G and thereafter centrifuged two additional times at 30 000 G for 30 min with the liquid phase poured off between each centrifugation. This was due to the Aspa samples having a very high water retention capacity, meaning that one round of centrifugation could not adequately separate the solid and liquid phases.

When only the solid phase remained in the bottle, an additional 200 ml of ultrapure water was added for the second leaching step (L/S 8). The samples were again shaken in an end-over-end tumbler for 24 h ± 30 min. The same centrifugation process as described above was repeated to obtain the liquid phase. The liquid phases were analysed for pH, electrical conductivity, alkalinity, anion concentrations and element concentrations. As experimental setup and all subsequent analyses were performed in open systems, measurement of dissolved oxygen and redox potential was excluded due to the risk for the measurements being affected by air. Due to the presence of sulfides in the solid samples^7^, it is assumed to be strongly reducing.

In addition, total elemental concentrations analysis and calculation of dry weight was performed on the solid phase on separate subsamples^7^.

### Electrical conductivity and pH

pH and electrical conductivity were measured using a Metrohm 744 pH meter and a Hach sensION+ EC7 meter, respectively. Calibration of pH meter was performed as a two point calibration using calibration solutions 7.01 and 10.01.

### Alkalinity

Alkalinity in the GLD leachate was measured by an endpoint auto titration unit using 0.02 M hydrochloric acid (HCl) as titrant on unfiltered leachate from samples (titrand). Endpoint was set to pH 5.4.

### Anions

Anions were analysed by an HP ^3D^CE capillary electrophoresis with a 50 cm, 40 μm capillary. A buffer made of chromate was used to condition the electrophoresis capillary both before and after each sample was analyzed. The chromate buffer consisted of 0.75 ml 0.1 M chromate mixed with 0.375 ml 20 mM TTAB (tetradecyltrimethylammonium bromide) and 13.875 ml ultrapure water.

### Element concentrations in the liquid phase

An aliquot of approximately 15 mL was allocated for elemental analysis. All leachate samples were filtered through 0.20 μm polytetrafluoroethylene (PTFE) syringe filters, acidified with sub-boiled nitric acid to 1% HNO₃ (v/v), and diluted 1:100 (v/v) with ultrapure water to mitigate ionization suppression from the saline matrix. Elemental concentrations were determined by inductively coupled plasma mass spectrometry (ICP-MS; Agilent 7500cx) for 29 elements in order of increasing m/z: ^7^Li, ^9^Be, ^23^Na, ^24^Mg, ^27^Al, ^39^K, ^43^Ca, ^51^V, ^53^Cr, ^55^Mn, ^56^Fe, ^59^Co, ^60^Ni, ^63^Cu, ^66^Zn, ^69^Ga, ^75^As, ^82^Se, ^85^Rb, ^88^Sr, ^95^Mo, ^107^Ag, ^111^Cd, ^125^Te, ^137^Ba, ^205^Tl, ^204+206+207+208^Pb, ^209^Bi, and ^238^U. Quantification used five- or six-point external calibration from ICP multi-element standard solution VI (Merck, Germany), with ranges adjusted to each analyte. Non-spectral interferences were corrected using ^103^Rh as an internal standard at 10 µg/L in all solutions. All measurements were conducted in triplicate, with tap water serving as an in-house reference solution to monitor method stability. For additional methodological description, see Zeiner et al.^15^.

Analytical precision (RSD) ranged from 0.15% to 2.2% for all analytes, except Bi and U (5–7%). Element recoveries in reference solution of 93–109% confirmed method trueness. LODs were < 0.001 µg/L for As, Co, Pb, Rb, Sr, Te, and U; 0.001–0.005 µg/L for Ag, Ba, Be, Bi, Cd, Ga, Mg, Mn, and Tl; 0.006–0.06 µg/L for Al, Cu, Fe, Li, Mo, Ni, and V; and up to 2.5 µg/L for Ca, Cr, K, Na, Se, and Zn.

### Correlation calculations and statistical descriptions

Correlation between parameters was calculated by Pearson correlation, using descriptions suggested by Evans^16^. When presenting statistical data average, median, 10^th^ percentile and 90^th^ percentile was chosen for data summary^17^. For calculation of amount leached out the total L/S 10 concentration in mg/kg d.w. was compared to total concentrations in solid GLD on a separate subsample as reported by Stahre et al.,^7^. In some cases, the leached concentration exceeded the total concentration in solid GLD. There are two major reasons causing leaching above 100% for a few elements. One group (Na, K, Rb) is due to very high concentrations and very high leachability (almost quantitative); exceeding 100% is caused by the analytical uncertainty for both the total concentration and the leachable concentration. Another group (As, Se, Mo, Bi) is caused by total concentrations close to the detection limit. However, there is one sample, Ös F18, with very high leaching for several elements that should be considered anomalous (perhaps due to heterogeneity of the samples). This sample and handling of it is further discussed in the “Results and discussion” section.

### Geochemical calculations

Geochemical calculations were performed using PHREEQC (with the Minteq.v4 database) to determine the possible presence of solid phases governing leachable concentrations through equilibrium^18^. Calculations were run as batch calculations with pH, alkalinity, anion concentrations and elemental concentrations. Temperature was set to 18.5° C (temperature in the laboratory). It was decided to initially set the redox potential (as pe) to -4 for all samples. This redox potential was selected as a compromise due to the presence of both sulfate (S(VI) and sulfide (S(-II)) in the solid phase^7^ and the addition of fully oxygenated water during leaching. The charge balance for the calculations is between − 21 and 0.3% (median 11%). Decreasing the redox potential further (to pe -8) changes the saturation indices slightly (Table 2). More importantly, saturation index for mackinawite (amorphous FeS) is closer to saturation. Colloidal mackinawite is supposed to be the mineral giving green liquor dregs it greenish shiny colour, indicating that it might be the mineral buffering the redox potential. Calcite is also closer to equilibrium at pe -8. Geochemical calculation results using pe -8 will be used in the following discussion. Charge balances above 20% are associated with samples with pH below 11.5, indicating the charge balances being related to the well-known conflict between pH and titrated alkalinity being higher than total alkalinity at highly alkaline systems^18^. Saturation index (SI) is the logarithm of the ratio between the ion activity product (IAP) and the solubility product (K_sp_) for the solid phase. A saturation index (SI) between − 0.5 and 0.5 was considered close to equilibrium, while a SI above 0.5 was regarded as supersaturated and a SI below − 0.5 was regarded as subsaturated. Ratios for each sample were also calculated by dividing the L/S 10 concentrations by the L/S 2 concentrations. A ratio < 2 was interpreted as equilibrium with a solid phase due to near constant leaching in both leaching steps. A ratio above 5 was interpreted as being dominated by a washing out process.

**Table 2:** Saturation indices for selected minerals and charge balances at pe -4 and pe -8.

	pe -4		pe -8	
	10^th^ percentile	90^th^ percentile	10^th^ percentile	90^th^ percentile
Charge balance (%)	-21.1	0.28	-5.5	31.3
**SI**				
Brucite (Mg(OH)_2_)	-1.4	0.51	-0.77	-1.4
Calcite (CaCO_3_)	-4.2	-1.0	0.09	1.2
Ferrihydrite (Fe(OH)_3_)	-1.7	1.7	-2.6	-0.29
Mackinawite (FeS)	-51	-24	-16	0.23
Pyrochroite (Mn(OH)_2_)	-2.7	1.2	-2.0	1.2
Rhodochrosite (MnCO_3_)	-2.7	0.58	-1.3	1.8
Gypsum (CaSO_4_*2H_2_O)	-7.6	-4.0	-5.3	-2.9

## Results and discussion

### pH, electrical conductivity and alkalinity

**Fig. 2:**
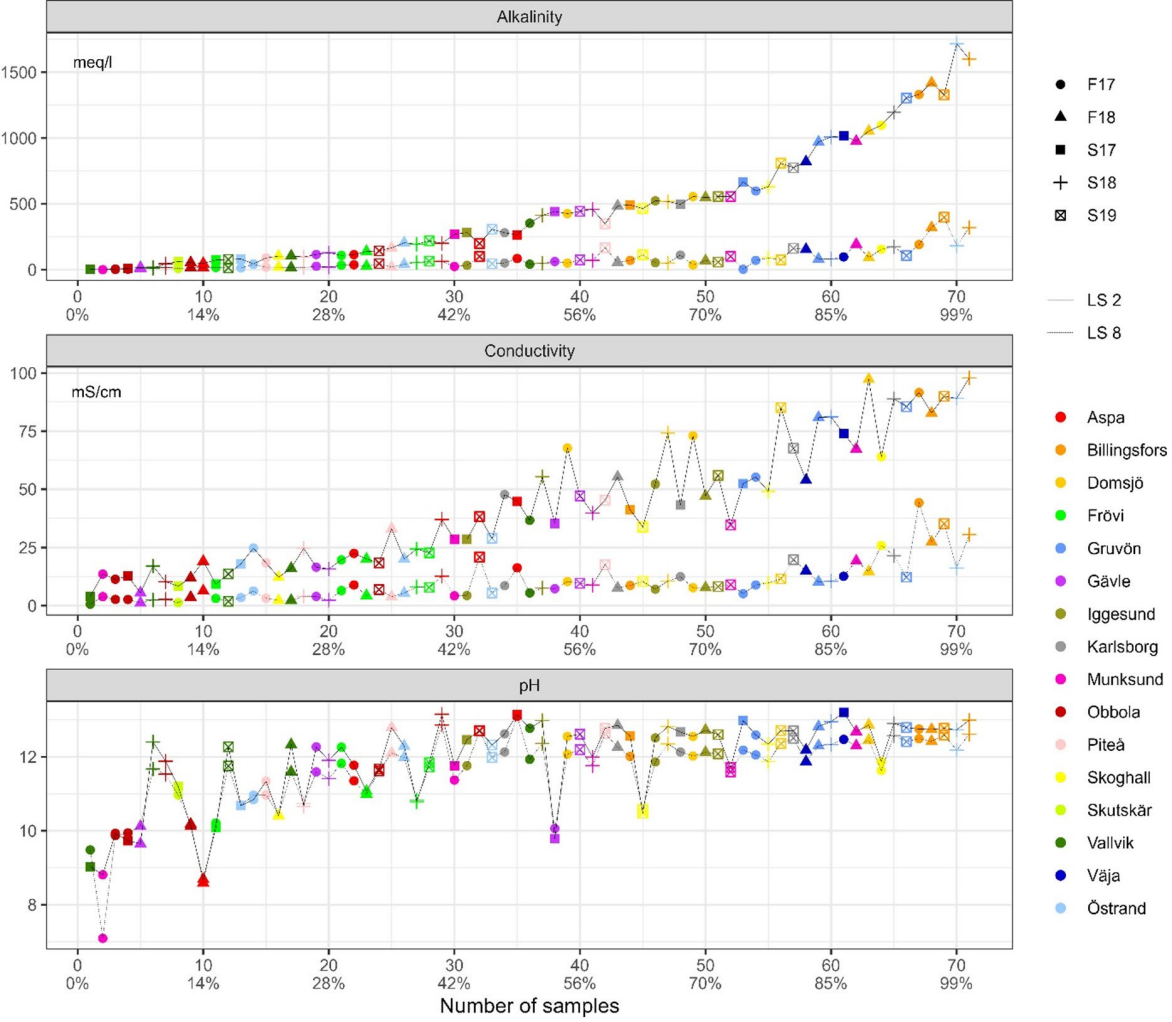
Alkalinity, electrical conductivity and pH divided by leaching step (L/S 2 and L/S 10). The lines between points are guidelines for separating which points are L/S 2 and which are L/S 10.

pH and electrical conductivity were, as expected, very high, Table 3; Fig. 2. In the first leaching step (L/S 2), pH is strongly correlated to electrical conductivity, but it was only moderately correlated in the second (L/S 10) leaching step. Electrical conductivity and alkalinity are generally strongly correlated to each other and to sodium and potassium concentrations, with the correlation being higher between the parameters from the same leaching step in comparison when compared to those between leaching steps. pH is most likely dependent on soluble hydroxide salts like NaOH and complexation. pH is in range with those reported by Jia et al.^3,8^ and Mäkitalo et al.^9^. Electrical conductivity on the other hand mostly depends on the soluble salt content. Alkalinity varied considerably between GLD from different mills at L/S 2, but was overall much lower at L/S 10, Table 3; Fig. 2. Alkalinity is suggested to depend on two parts: easy soluble salts and long-term buffering capacity. The easy soluble salts (mainly NaOH) provide instant buffering, but are consumed in the process, while the long-term buffering capacity (mainly provided by CaCO_3_) is slow yet capable of buffering large volumes of acid leachates.

**Table 3:** A pH, electrical conductivity (mS/cm) and alkalinity (meq/l) in the two different liquid to solid leaching ratios (L/S 2 and L/S 10), *n* = 71.

Leaching step	pH	pH	Electrical Cond.	Electrical Cond.	Alkalinity	Alkalinity
	L/S 2	L/S 10	L/S 2	L/S 10	L/S 2	L/S 10
10^th^ percentile	10.08	10.18	12.2	2.45	44.6	13.2
Average	11.93	11.60	42.4	9.75	460	73.0
Median	12.40	11.87	37.0	7.77	349	50.4
90^th^ percentile	12.90	12.50	85.1	19.8	1 100	167

### Anions

Sulfate $({\mathrm{SO}}_{{\mathrm{4}}}^{{{\text{2 - }}}} {\mathrm{)}}$ was the most commonly occurring anion, along with carbonate (alkalinity), Table 4. Sulfate concentrations decreased significantly between the first and the second leaching step, Table 4, indicating that this anion is part of the water soluble salts that are dissolved and washed out primarily in the first leaching step. Gypsum (CaSO_4_*2H_2_O) does not seem to be controlling sulfate concentrations as saturation indices are clearly below saturation (saturation indices between − 5.7 and − 2.9). Low sulfate concentrations also support the assumption of reducing conditions in the samples. Chloride does occur in the samples, but the margin for error is large, as the peak for phosphate is often so large that it interferes with the peak for chloride during analysis. Nitrate and fluoride occur sporadically above the quantification limit, Table 4. No clear correlation for different mills can be seen for any of the anions, however nitrate seems to mainly occur in the two most northern and the three most southern mills, while fluoride is mainly found in the four most northern mills. Nitrate is suggested to be derived from the wood as there are no other large source of nitrogen in the cooking process while sulfate is derived from the cooking process as sodium sulfate (Na_2_SO_4_) is used as a cooking chemical. The source of chloride and fluoride is believed to be contamination of water used in the washing and cooking chemicals, but this has not been ascertained. In samples that contain lime mud, carbonate has been detected but not quantified. However, the results from the alkalinity analysis above suggests that the carbonate concentration is significant.

**Table 4:** Anions (mg/l) in the two different liquid to solid leaching ratios (L/S 2 and L/S 10), *n* = 71.

Leaching step	Cl		SO_4_		NO_3_		F	
	L/S 2	L/S 10	L/S 2	L/S 10	L/S 2	L/S 10	L/S 2	L/S 10
10^th^ percentile	12.5	3.14	411	69.6	356	62.4	30.2	6.82
Average	435	48	2 360	649	849	212	118	217
Median	93.8	22.0	1 190	267	847	76.3	155	42.9
90^th^ percentile	426	72.1	5 060	1 260	1 540	475	166	214
No. Samples < DL	45	45	2	2	55	58	63	60

### Elemental leaching

**Fig. 3:**
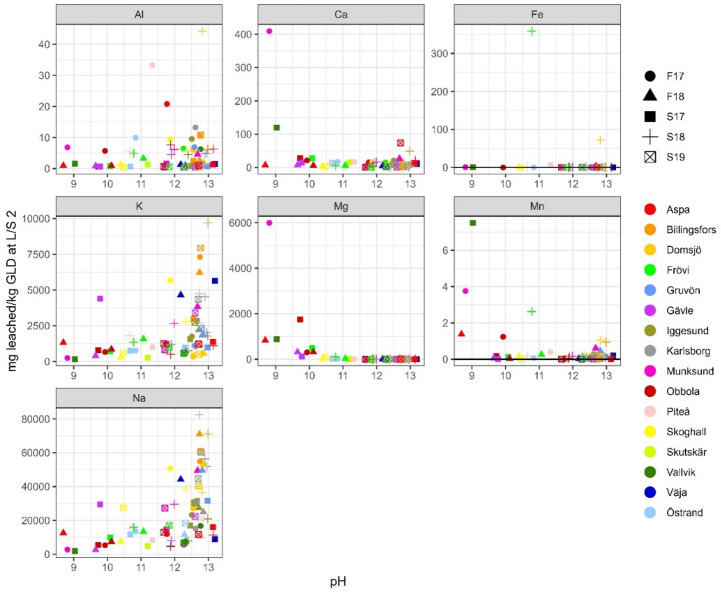
Leached L/S 2 concentrations (in mg/kg d.w.) for major elements plotted against pH, *n* = 71. If any sample was below detection limit for an element the detection line has been added to the plot.

**Fig. 4:**
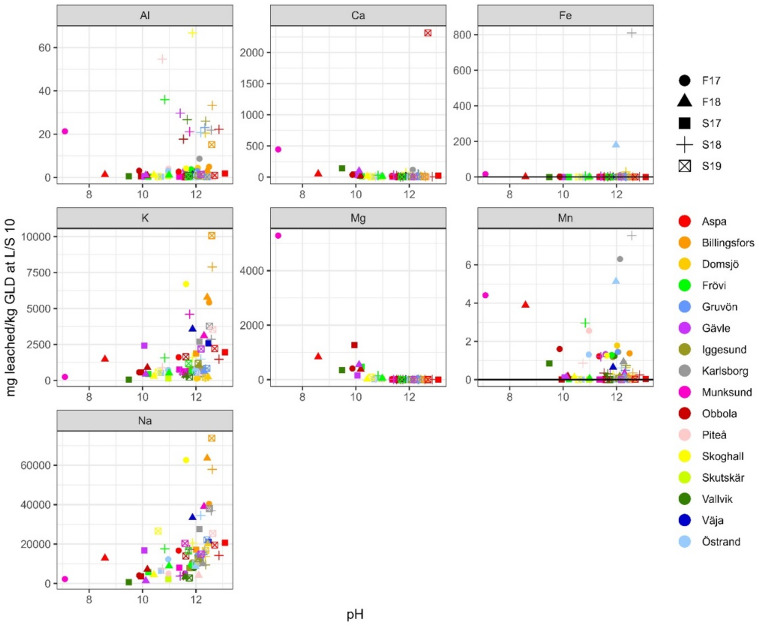
Leached L/S 10 concentrations (in mg/kg d.w.) for major elements plotted against pH, *n* = 71. If any sample was below detection limit for an element the detection line has been added to the plot.

**Fig. 5:**
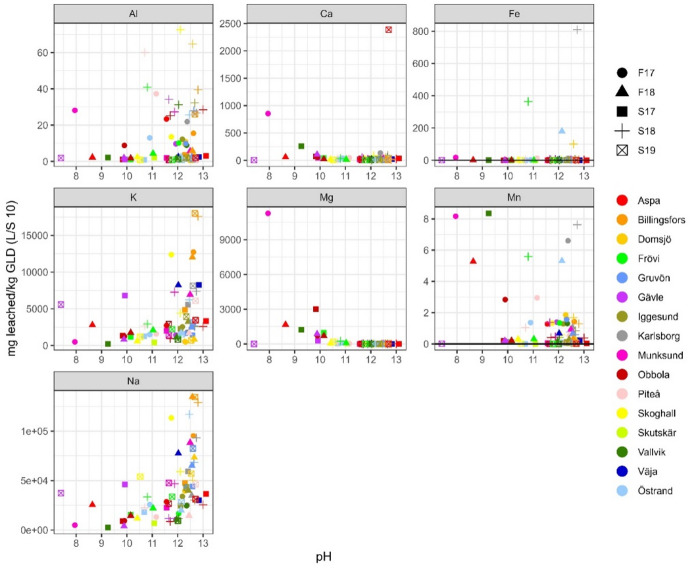
Leached L/S 10 concentrations (in mg/kg d.w.) for major elements plotted against average pH, *n* = 71, plotted against pH calculated as an average from pH at L/S 2 and L/S 8. If any sample was below detection limit for an element the detection line has been added to the plot.

Sodium and potassium are the most leachable major elements. When comparing the amount leached at L/S 10, Fig. 5, to the total amount in GLD (presented in^7^), an average of 41 500 mg/kg d.w. of sodium (corresponding to 71% of sodium being leached out), and 3 500 mg/kg d.w. of potassium (76%) were leached out. This is generally in congruence with what other researchers have reported, Table 5. There is, however, a difference in the number of samples behind the data. Previous studies cover only a few or one sample while this study covers 71 samples. When looking at the ranges of values of previous research, results indicate that GLD is very heterogeneous with large variation in leached concentration of the major elements. However, when comparing previous results to results in this study, the range for sodium and potassium becomes less pronounced with less extreme values. This shows that even if there can be large differences between some samples, there is a cohesion when looking at GLD as a larger population, i.e. there is more homogeneity than the previous, small sample studies, indicated. The results also indicate that the amount of cooking chemicals that cannot be recovered are approximately the same regardless of mill or chemical recovery process. While this needs further study to verify it proposes that the chemical recovery of cooking chemicals is limited by extraction kinetics. When plotted against pH, Figs. 3, 4 and 5, both sodium and potassium also show an apparent correlation, where higher pH corresponds to a higher leachable amount. This is however not a pH dependence but a covariation between sodium ions and hydroxide ions, as the main origin of both is the cooking chemicals, consisting of NaOH and Na_2_SO_3_ (where potassium occurs as a contaminant), that have not been properly washed out due to inefficiency of the wash cycle. Geochemical calculations indicate only natron (Na_2_CO_3_) as a solubility controlling phase at extremely high sodium concentrations. Pirssonite (Na_2_Ca(CO)_3_*2H_2_O) is not in the database used, but several studies^19–22^, have identified pirssonite as one of the Na-minerals occurring in GLD. Therefore, a more thorough geochemical calculations including pirssonite may yield a different result. The calculated Pearson correlation factors between pH and these two elements vary from moderate to weak, indicating that these two elements are present as easily soluble salts. Seen from an environmental point of view, the extremely high sodium concentrations leached in this study can be of concern. Several studies^23–25^ have found that increased leaching of sodium has resulted in increased trace element mobility, mostly due to cation exchange.

**Table 5:** Reference values as reported by other researchers for batch leaching test LS 10, “-” indicates that no value was reported by author whereas “NA” are not analysed.

LS 10 mg/kg	This study	Jia et al.,^8^	Jia et al.,^3^	Mäkitalo et al.,^9^
Country of origin	Sweden	Sweden	Sweden	Sweden
Number of samples	71 from 16 mills	1 from 1 mill (3 replicates)	7 from 5 mills	1 from 1 mill (3 replicates)
pH	11.7 ± 1.19	12.2 ± 0.2	9.79–12.9	-
Al	12.4 ± 16.1	0.08	3.29–32 800	0.34 ± 0.19
Ca	77.7 ± 294.7	0.003	6.89–90.3	29.4 ± 18.0
Fe	32.8 ± 133.9	0.0003	0.053–0.434	0.23 ± 0.00
K	3 540 ± 3 740	< 750	102–24 100	1 430 ± 12
Mg	307 ± 1 390	0.0001	77.6–2 540	8.3 ± 3.0
Mn	1.32 ± 2.02	0.003	0.0038–0.36	0.97 ± 0.76
Na	41 500 ± 32 400	NA	8 200–226 000	-
As	0.071 ± 0.068	1.9	0.02–0.51	0.002 ± 0.002
Cd	0.020 ± 0.024	0.01	0.0002–0.008	0.0003 ± 0.0000
Co	0.012 ± 0.015	-	0.00062–0.0063	0.0006 ± 0.0000
Cr	1.93 ± 6.30	71.7	0.01–3.64	0.44 ± 0.07
Cu	2.33 ± 7.00	0.2	0.02–0.16	0.045 ± 0.015
Mo	0.884 ± 2.23	NA	51.6–2 510	-
Ni	0.686 ± 3.24	0.2	0.01–0.06	7.01 ± 3.00
Pb	0.114 ± 0.136	0.008	0.0–0.0	1.89 ± 0.00
Zn	1.86 ± 4.12	0.03	0.18–8.81	0.088 ± 0.051

All values except pH are in mg/kg d.w. Values for this study are reported as average value ± standard deviation. The sample reported in Jia et al.^8^ is from a sealing layer on a 5 year old tailings landfill, while the samples reported in Jia et al.^3^ are for fresh GLD.

When looking at high sodium concentration in alkaline leachate from GLD that is mixed with acid rock drainage, it is questionable if the possibility of increased trace element mobility effect is strong enough to be of concern. More likely, as pH is stabilised to near neutral when mixing ARD and GLD, trace element mobility decreases in the runoff due to the increase in pH. Regardless, any effect from the cation exchange effect will quickly decrease with time as sodium is easily washed out from the GLD. It is also possible that the extremely high sodium concentrations present in this study do not create the cation exchange effect. Studies done by Pontoni et al.,^26^ showed that increased sodium concentrations decrease the cation exchange effect and the sodium concentrations in this study greatly exceed those presented in Bäckström et al.,^23^, Löfgren^24^ and Norrström and Bergstedt^25^.

**Fig. 6:**
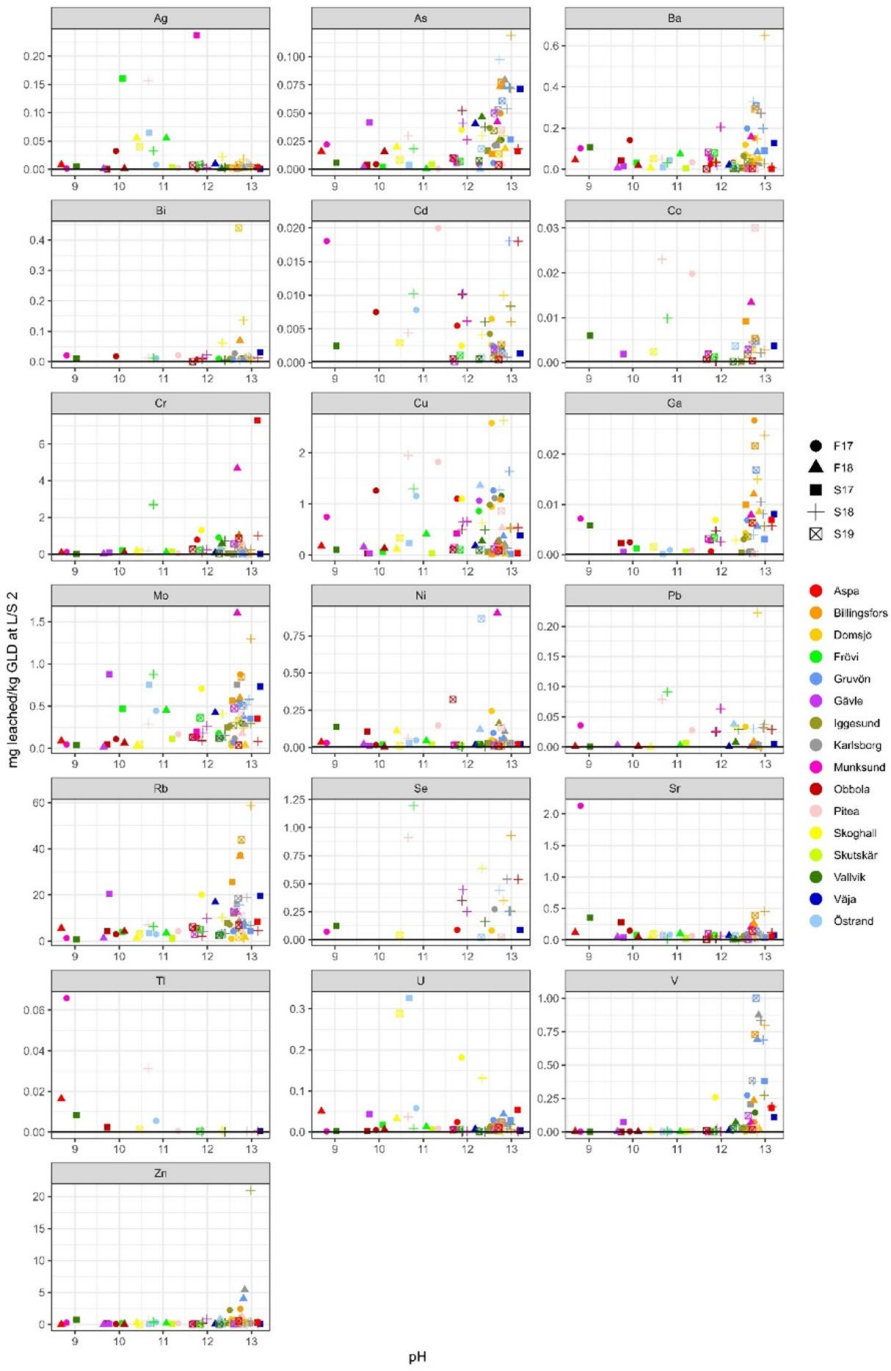
Leached L/S 2 concentrations (in mg/kg d.w.) for trace elements plotted against pH, *n* = 71. If any sample was below detection limit for an element the detection line has been added to the plot.

**Fig. 7:**
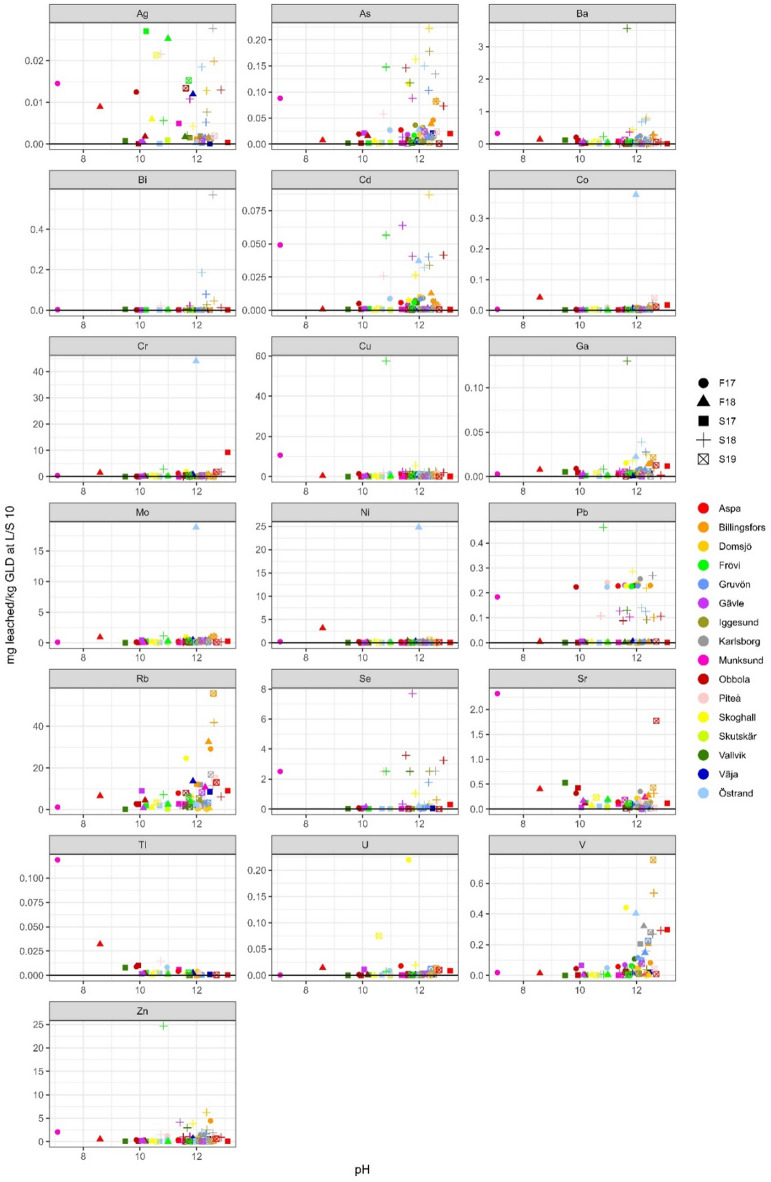
Leached L/S 10 concentrations (in mg/kg d.w.) for trace elements plotted against pH, *n* = 71, If any sample was below detection limit for an element the detection line has been added to the plot.

**Fig. 8:**
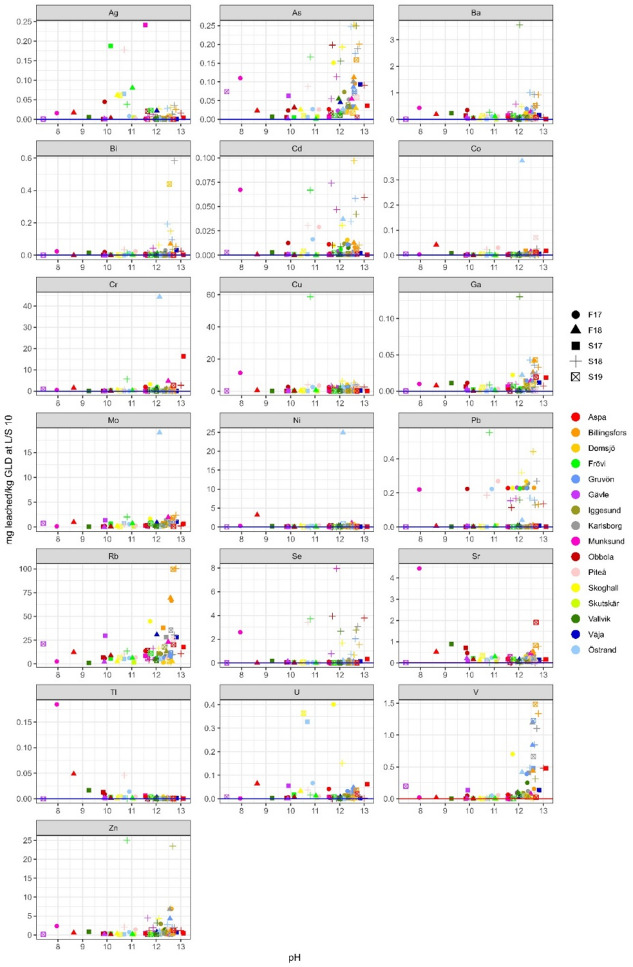
Leached L/S 10 concentrations (in mg/kg d.w.) for trace elements plotted against pH calculated as an average from pH at L/S 2 and L/S 8, *n* = 71. If any sample was below detection limit for an element the detection line has been added to the plot, for samples below detection limit at LS 2 the line is red, and for samples below the detection limit at LS 8 the line is blue.

Rubidium has a similar leaching profile as sodium and potassium and is generally strongly correlated according to Pearson correlation calculations. This is expected as the origin of rubidium is almost solely as a contaminant in potassium chemicals^7^. As such it also shows a clear pH dependence, Figs. 6, 7 and 8, and is also the trace element that leached the highest concentration of all trace elements. Rubidium has a low average total concentration in GLD but leached on average 15.8 mg/kg d.w. (63%).

Calcium on the other hand is, on average, the most abundant element in GLD, ranging from 4.9% to 38% d.w^7^. Calcium leaching was low, Figs. 3, 4 and 5, but at a higher rate in this study compared to what is reported by others, Table 5. The main reason for the differences in results is the type of samples. The samples in Jia et al.,^3^ are mainly from mills that do not add lime mud during dewatering, while the sample in Jia et al.,^8^ is from a sealing layer that is five years old at the Rönnskärsverket in Skellefteå. This is a location with high background acidification due to smelting, thus it is highly probable that calcium carbonates in the sample have dissolved and calcium have leached out. On average calcium leaching was 0.03% when excluding the one extreme sample (Aspa S19) that leached 1.86%, and 0.05% when including the extreme sample. The ratio between calcium leaching in L/S 2 and L/S 10 is low. This indicates that unlike sodium and potassium, calcium is mainly solubility controlled. Geochemical calculations suggests calcium ions are in equilibrium with calcite (CaCO_3_) (SI 0.1 to 1.2 (percentile 10–90)), which is congruent with the main source of calcium in GLD being CaCO_3_ in the lime mud used for dewatering. Prescence of calcite has been confirmed in several mineralogical studies^19,9,22^. In general, calcite is the only mineral identifiable in mineralogical studies due to GLD being mainly amorphous. Due to the low calcite (CaCO_3_) solubility and slow dissolution rate, it has little effect on short time buffering capacity compared to the dissolved hydroxides. However, over longer periods of time as carbonate is consumed by acid from the sulfuric mine waste it is likely that a steady state dissolution will occur, resulting in a long term alkalinity.

Aluminium and manganese leaching were also very low, on average 0.32% for aluminium and 0.01% for manganese. Manganese did not show any pH dependence whereas aluminium appeared to have a weak pH dependence on leaching behaviour, Figs. 3, 4 and 5. Aluminium leaching is in range with what is reported by other researchers, with the exception of two samples reported by Jia et al.,^3^. It is probable that the pH dependence for aluminium is due to complexation with soluble hydroxides. L/S 2 and L/S 10 ratios, Figs. 3, 4 and 5, indicate that manganese is in equilibrium, and geochemical calculations suggest pyrochroite (Mn(OH)_2_) (SI -2.0 to 1.2)) and rhodochrosite (MnCO_3_) (SI 1.3 to 1.8)).

Magnesium and iron generally had low leaching, but both have a few extreme samples, Figs. 3, 4 and 5, where the leached amount corresponds to up to 20% of the total concentration in GLD, Table 6. Of 42 samples with iron concentrations above detection limit, 28 leached < 0.1% and only 3 samples leached > 1%; however, one of those samples, Karlsborg S18, is an extreme that leached 20.0%. For magnesium, 55 of 71 samples leached < 0.1% and 8 samples leached > 1%. The two samples with the highest dissolved concentrations, Munksund F17 and Vallvik S17, are odd samples that leached 11.8% and 18.6%. Like calcium and manganese, magnesium and iron concentrations (excluding the extreme samples for iron) are most likely controlled by equilibrium as indicated by the L/S 2 and L/S 10 ratios, Figs. 3 and 4. Geochemical calculations suggest equilibrium primarily with brucite (Mg(OH)_2_) for magnesium (SI -0.8 to 1.4) and ferrihydrite (SI -2.5 to -0.3) for iron. Brucite has indeed been detected in a few mineralogical studies in GLD^19,9^. In this study, concentrations of Fe, Mg and Mn seem to be higher than previously reported by other researchers. However, when only comparing samples from the mills used by both this study and previous research it can be observed that the compared samples are in the similar range or lower. This effect is most pronounced when looking at the Jia et al.,^3^ study that has the most samples of previous research. Approximately half of the samples in this study were either below detection limit or higher than the samples in the Jia et al.,^3^ study despite being from the same mills. This clearly shows the need for a large sample population study when determining GLD characteristics, due to the inherent heterogeneity of GLD small sample studies do not show the full picture.

**Table 6:** Minimum, average and maximum for leached (L/S 10) concentrations (both as mg/kg d.w. and %) for major elements.

	Al	Ca	Fe	K	Mg	Mn	Na
Leached (mg/kg d.w.), 10^th^ percentile	0.907	3.75	0.094	772	0.809	0.041	9 390
Leached (mg/kg d.w.), average	12	78	32.8	3 540	307	1.32	41 500
Leached (mg/kg d.w.), median	3	25	2.49	2 190	8	0.59	33 500
Leached (mg/kg d.w.), 90^th^ percentile	32.3	68.4	12.6	8 140	692	4.34	88 500
Samples > DL	71	71	42	71	71	65	71
Leached (%.), 10^th^ percentile	0.014	0.002	0.003	43.0	0.003	0.00019	40.9
Leached (%), average	0.32	0.05	0.92	75.6	0.86	0.01	71.2
Leached (%), median	0.11	0.01	0.05	78.4	0.02	0.00	75.3
Leached (%.), 90^th^ percentile	0.941	0.035	0.418	104	2.20	0.027	91.3
Samples > DL	71	71	42	71	71	65	71
Ratio, 10^th^ percentile	0.31	0.69	0.14	0.38	0.65	0.27	0.39
Ratio, average	1.65	4.22	187	0.75	2.38	7.65	0.72
Ratio, median	0.76	1.93	2.21	0.63	2.13	2.07	0.60
Ratio, 90^th^ percentile	4.53	7.40	18.1	1.27	4.78	22.76	1.22
Samples > DL	71	71	42	71	71	65	71

Ratios are calculated as L/S 10 concentrations divided by L/S 2 concentration for each sample. Please note that the total concentrations and leached concentrations minimum and maximum are not necessarily from the same samples. For concentrations in solid GLD please see Table 2 in Supplementary data.

Ag, Cd, Co, Cu and Pb leaching was on average very low, leaching, 0.03, 0.02, 0.01, 2.33 and 0.11 mg/kg d.w., respectively, corresponding to 0.54%, 0.40%, 0.16%, 1.46% and 1.41% with a few extremes, Table 7. Arsenic on the other hand leached a moderate amount of 0.07 mg/kg, corresponding to 33.0%, Table 7. For these elements, copper was the only element with all sample concentrations above detection limit. The Frövi S18 sample, with the highest leached concentration of copper (58.8 mg/kg d.w. corresponding to 28.3%), is also the sample with the highest leached concentration of lead (0.55 mg/kg d.w. corresponding to 1.3%), Figs. 6, 7 and 8. L/S 2 and L/S 10 ratio comparison and geochemical calculations suggest that lead is partly in equilibrium with Pb(OH)_2_ (SI -3.0 to 0.7), Table 7. No clear pH dependence can be seen for Ag, Cd, Cu and Pb, but a weak pH dependence can be seen for arsenic and cobalt. When comparing samples from the mills analysed in both this study and previous studies it can be observed that the compared samples vary between elements. Copper is the only element of the above mentioned elements where this study had higher leached amounts than all three previous studies. But when comparing the leached amount of copper between the three studies there is a noticeable difference between the reported values. This again shows the importance of a large sample population when characterisation of a heterogeneous material such as GLD is done.

**Table 7:** Minimum, average and maximum for leached (L/S 10) concentrations (both as mg/kg d.w. and %) for minor elements.

	Ag	As	Ba	Bi	Cd	Co	Cr	Cu	Ga	Mo	Ni	Pb	Rb	Se	Sr	Tl	U	V	Zn
Leached (mg/kg d.w.), 10^th^ percentile	0.002	0.009	0.012	0.009	0.001	0.003	0.094	0.104	0.001	0.148	0.034	0.003	2.408	0.469	0.052	0.003	0.003	0.041	0.140
Leached (mg/kg d.w.), average	0.030	0.071	0.235	0.055	0.020	0.012	1.93	2.33	0.014	0.884	0.686	0.114	15.8	2.45	0.315	0.033	0.039	0.229	1.86
Leached (mg/kg d.w.), median	0.013	0.042	0.109	0.022	0.010	0.005	0.435	0.475	0.010	0.473	0.138	0.039	8.71	2.58	0.169	0.013	0.011	0.055	0.650
Leached (mg/kg d.w.), 90^th^ percentile	0.064	0.185	0.514	0.111	0.059	0.026	12.6	3.47	0.034	1.351	0.487	0.269	30.6	3.84	0.538	0.062	0.064	4.34	4.30
Samples > DL	44	64	70	28	42	21	54	71	46	71	58	35	71	17	62	10	55	69	71
Leached (%), 10^th^ percentile	0.028	4.53	0.003	9.67	0.022	0.015	0.061	0.051	0.023	23.6	0.049	0.034	35.4	-	0.018	0.275	1.000	0.148	0.006
Leached (%), average	0.537	33.0	0.070	252	0.404	0.159	1.73	1.46	0.341	112	1.26	1.41	63.1	-	0.087	7.21	5.97	0.224	0.163
Leached (%), median	0.277	22.8	0.026	61.6	0.166	0.091	0.518	0.446	0.149	62.8	0.324	0.302	66.1	-	0.048	2.50	3.67	0.224	0.036
Leached (%), 90^th^ percentile	1.19	89.7	0.149	763	0.820	0.377	3.18	2.78	0.948	107	1.79	4.44	91.01	-	0.122	19.2	13.4	0.300	0.329
Samples > DL	44	48	70	20	42	21	54	71	46	71	58	35	71	0	62	10	49	2	71
Ratio 10^th^ percentile	0.12	0.20	0.43	0.10	0.45	0.21	0.40	0.23	0.40	0.35	0.45	0.26	0.38	0.59	0.74	0.92	0.03	0.26	0.29
Ratio, average	1.21	5.42	19.1	3.68	2.86	2.73	5.74	2.43	1.92	3.56	10.0	83.2	0.71	7.56	1.84	2.81	0.29	4.17	5.01
Ratio, median	0.61	0.66	1.28	0.24	1.49	0.98	1.19	1.07	1.14	0.80	1.67	2.49	0.61	2.75	1.60	1.87	0.19	0.91	1.34
Ratio, 90^th^ percentile	2.97	4.26	7.38	5.32	6.38	4.23	7.84	3.77	3.33	2.35	14.1	9.09	1.22	21.7	2.54	6.19	0.67	9.05	12.2
Samples > DL	44	64	70	28	42	21	54	71	46	71	58	35	71	17	62	10	55	69	71

Ratios are calculated as L/S 10 concentrations divided by L/S 2 concentration for each sample. Please note that the total concentrations and leached concentrations minimum and maximum are not necessarily from the same samples. For selenium, the three samples with Se > DL for total concentrations in GLD are not the same as the 17 samples with leached concentrations > DL. For concentrations in solid GLD please see Table 3 in Supplementary data.

Cr, Mo and Ni generally leach low concentrations (on average 1.93, 0.88, and 0.69 mg/kg d.w.) and they all have the same sample, Östrand F18, that stands out, Figs. 6, 7 and 8; Table 7. Chromium concentrations are lower than those reported by Jia et al.,^8^, in range with Jia et al.,^3^ and slightly higher than Mäkitalo et al.,^9^. Nickel concentrations were lower than those reported by Mäkitalo et al.,^9^, but in range with what is reported by Jia et al.,^8^ and Jia et al.,^3^. The differences between the ranges in the three previous studies is most noticeable for chromium, reiterating the need for a larger sample population study in order to find general characteristics for a heterogeneous material such as GLD. L/S 2 and L/S 10 ratio comparison, Figs. 6 and 7, suggest that nickel is partly in equilibrium, but geochemical calculations was inconclusive regarding what minerals the equilibrium is with. Disregarding the sample Östrand F18, chromium and nickel leached on average ≤ 1% of total concentration in GLD. The reason for the exclusion of sample Östrand F18 is due to an odd behaviour of iron, nickel, molybdenum and chromium in this sample. For example, molybdenum leached 3 400% according to leaching calculations, Table 7. In the first leaching step, as well as the elemental analysis in solid GLD done by Stahre et al.,^7^ the concentrations appear normal and in range with what is expected based on the behaviour of both the whole sample population, as well as other samples from this mill. But, in the second leaching step the concentrations of these three elements are significantly higher than what is reasonable. There can be two explanations for this; A contamination occurred during handling of the sample post first leaching step. Or the sample contained a grain of a mineral high in these three elements that was dissolved during the second shaking period due to physical breakage. It is not uncommon to find foreign grains in GLD, but it is uncommon that it results in this type of noticeable elevated concentrations when no visible grain is found. However, the Östrand mill have had major renovation on its process line during the ongoing of this study so there is a reasonable risk of contamination from breaking in new equipment.

Molybdenum is easily leached, leaching on average 65.5% (excluding the sample Östrand F18), and has leached up to and above 100% in several samples despite low leaching concentrations. Leaching > 100% is impossible, but leached concentrations slightly above 100% can be attributed to analytical uncertainty. It is apparent that although molybdenum is easily leached, there is a disparity in the analysis of molybdenum in leachate and analysis of solid material. It is also probable that molybdenum is unevenly distributed in solid GLD, resulting in samples being heterogenic as they go into analysis despite homogenisation. Geochemical calculations suggest that molybdate (MoO_4_^-^ likely in the form of CaMoO_4_ *(s)*) is the main solid species for molybdenum. Molybdenum concentrations in this study were lower than those reported by Jia et al.,^3^.

Leaching of zinc was originally a concern, as total zinc concentrations in GLD are high, with an average of 2 100 mg/kg d.w. and up to 6 000 mg/kg d.w^7^. However, when looking at the leaching data for zinc, total leaching was on average only 0.16% (1.86 mg/kg d.w.) with a median of 0.04% (0.65 mg/kg d.w.), Figs. 6, 7 and 8; Table 7. When comparing with previous research concentrations, this study is in range with Jia et al.,^3^ and higher than the other two studies. L/S 2 and L/S 10 ratio comparison and geochemical calculations suggest that zinc is partly in equilibrium with Zn-oxides (zincite (Zn, Mn)O, Zn(OH)_2_(gamma) and ZnO(active)). A concern has been raised about using GLD for mining waste remediation due to relatively high total zinc concentrations^7^, typically already high in the effluent from ARD. However, this study shows that zinc leaches very little from GLD and therefore high total zinc concentrations in GLD are not prohibitive for use in acid mining waste remediation.

Leaching of barium, gallium, uranium, thallium, strontium and vanadium was generally low, on average 0.24 mg/kg d.w. (0.07%), 0.014 mg/kg d.w. (0.34%), 0.039 mg/kg d.w. (6.0%), 0.033 mg/kg d.w. (7.2%), 0.32 mg/kg d.w. (0.087%) and 0.23 mg/kg d.w. (0.2%), Figs. 6, 7 and 8; Table 7. L/S 2 and L/S 10 ratio comparison and geochemical calculations suggest that barium is partly in equilibrium with barite (BaSO_4_) while strontium is partly in equilibrium with strontianite (SrCO_3_). Strontium also has a somewhat similar leaching profile as calcium.

For selenium the leaching ratio cannot be determined as only two samples were above DL in the solid material analysis. DL for the analysis of selenium in the spring 2018 batch was significantly lower that DL for analysis of the other batches, Figs. 6, 7 and 8. In general, it can be concluded that selenium is seldom present in GLD. Only 20 samples had bismuth concentrations above DL for both solid GLD and leachate, and bismuth leached very inconsistently between samples, Figs. 6, 7 and 8; Table 7. Three samples leached less than 10%, nine samples leached 10–100% and eight samples leached > 100%. In the remaining samples, at least one of the leachate or solid material analysis, or both, were below detection limit rendering comparison impossible.

Leaching of trace elements is thus fairly low and lowering the pH to a more neutral pH will most likely decrease it further. However, while trace element leaching due to high pH in general will decrease when pH is adjusted toward neutral^2,3^, this is not certain. While leaching with only water, as was the case in this study, gives a good insight into the basic leaching properties of GLS and is a good indication of suitability for usage as a remediant, it does not give the full picture. Other factors such as presence of sulfur and carbonates, redox conditions, element speciation etc. can have a high impact on leachability. Therefore, additional studies are needed for verification on the leaching behavior of GLD in different geochemical environments and its suitability for remediation.

When comparing the results in this study to the limit values, Table 8, it is observed that all samples leached below or well below the leaching limit for As, Ba, Cd, Pb and Zn. For Cr, Cu, Mo and Ni only one sample leached above the limit values. For Cu this sample was Frövi S18 and for Cr, Mo and Ni it was the unusual outlier sample Östrand F18 that has been discussed above. For selenium only two samples of the 17 that had concentrations above detection limit leached less that the leaching limit.

**Table 8:** The Swedish concentration limits for non-hazardous waste for landfilling that is a direct implementation of the European waste disposal directive (1999/31/EG) into Swedish law.

Element	C_0_ (L/S = 0.1) mg/l	L/S = 10 mg/kg d.w.
As	0.3	2
Ba	20	10
Cd	0.3	1
Cr	2.5	10
Cu	30	50
Hg	0.03	0.2
Mo	3.5	10
Ni	3	10
Pb	3	10
Sb	0.15	0.7
Se	0.2	0.5
Zn	15	50

While the leaching limit values specified in Table 8 are not directly applicable in a remediation situation, they can be used as an indicator as to the overall suitability from a leaching perspective.

## Conclusions

Leaching of Ba, Ca, Fe, Mg, Mn, Ni, Pb, Sr, and Zn from green liquor dregs (GLD) is mainly controlled by mineral solubility and kinetic dissolution rate, whereas K, Na, Rb and U are mainly present in GLD as easily soluble salts that are easily washed out. Al, Ag, As, Bi, Cd, Co, Cr, Cu, Ga, Mo, Se, Tl and V are present in several forms with solubilities ranging from low to high.

Sodium (and potassium to some extent) is strongly correlated to electrical conductivity and alkalinity, indicating that the Na-salts are in the form of soluble oxides/hydroxides that act as the main short time buffer until consumed or washed out. Calcite remains in GLD, giving GLD a long-term buffering capacity that is not washed out, which is a necessity for applications related to acid neutralization.

Cd, Cr, Cu, Ni and Zn were originally of concern for both landfilling and in applications towards mining waste remediation, because their concentration in landfilling material and waste used for co-disposal is regulated in Sweden. These elements are also usually already present in high concentrations in mine waste effluents and remediation involves decreasing these trace element loads rather than increasing them. These elements were shown by Stahre et al.,^7^ to be present in GLD in total concentrations that could be problematic from a remediation point of view. However, the present study shows that on average leaching of these elements are very low to low. They are therefore not a hindrance in using GLD for acid rock remediation as these elements are relatively immobile.

For the other trace elements, it is likely that there will be a first flush effect due to soluble salts dissolving. After the initial flush, leaching is controlled by the amount of water flowing through the system. As it takes a long time to achieve a leaching rate of L/S 10 it is probable that the actual leached concentrations of trace elements will be lower than reported in this study.

In conclusion, the sample population studied is large enough to determine large scale variability and has thus addressed the main aim of the study. The results indicate that GLD is well suited to be used for treatment of acidic mining waste instead of being landfilled in non-hazardous landfills. This is a big step towards solving two considerable industrial waste problems in Sweden, as well as also globally: the remediation of many small, old, orphaned sulfidic mining sites, and the large-scale utilization of GLD at a reasonable cost.

### Future perspective

The environmental load from each respective waste will most likely decrease if the amount of GLD injected or otherwise mixed into the acidic waste is leveraged so that a more neutral pH is achieved. Leaching from the alkaline waste will decrease due to a loss of soluble hydroxy complexes as pH decreases, while leaching from the mining waste will decrease due to less aggressive weathering due to acid dissolution of minerals as pH increases to near neutral. When comparing the leached concentrations from GLD with concentrations from mining waste, there is an environmental benefit with using GLD as a remediant even if it is not inert. However, further studies are needed to verify this.

Future studies for the verification of usage of GLD as an acid mine waste remediant should include a leaching speciation test, i.e. a kinetic test. Also needed is a leaching test with different types of leachants for the purpose of observing the effect of acid speciation and presence of sulfur, or more specifically sulfate, and iron. These elements are very common in ARD and can have a notable effect on leaching properties. A long term buffering capacity test is also of interest to give a general calculation on how much GLD is needed for remediation of any given site.

## Supplementary Information

Below is the link to the electronic supplementary material.

Supplementary Material 1

## Data Availability

Data is provided within the manuscript and supplementary data file will be provided upon request.
