# Supplementary material for: Element leaching from green liquor dregs from 16 Swedish pulp and paper mills between 2017 and 2019
Source: Sci Rep. 2026 May 9;16:14683. doi: 10.1038/s41598-026-51421-1 (PMC13157502; doi:10.1038/s41598-026-51421-1)
Supplement: Supplementary file 1 — Supplementary Material 1 [file 41598_2026_51421_MOESM1_ESM.docx]

# Supplementary data

Table 1 Dry matter content in solid GLD, used for calculation of amount of wet sample needed in order to obtain a sample weight corresponding to 25 g dry sample for leaching (Stahre et al., 2024).

| **Dry Matter Content (%)** | | | | | | |
| --- | --- | --- | --- | --- | --- | --- |
|  | Spring 17 | Fall 17 | Spring 18 | Fall 18 | Spring 19 | Average |
| Aspa | 31.9 ± 0.3 | 29.8 ± 0.5 | 32.0 ± 0.7 | 32.8 ± 0.7 | 35.8 ± 0.3 | 32.5 ± 2.0 |
| Billingsfors | 35.2 ± 0.6 | 32.2 ± 0.3 | 28.1 ± 0.1 | 36.3 ± 0.2 | 29.2 ± 0.1 | 32.2 ± 3.2 |
| Domsjö | No sample | 49.0 ± 0.5 | 60.0 ± 2.4 | 52.9 ± 0.4 | 55.6 ± 2.6 | 54.4 ± 4.0 |
| Frövi | 47.0 ± 0.3 | 34.4 ± 0.2 | 37.2 ± 0.3 | 40.7 ± 0.5 | 36.3 ± 0.2 | 39.1 ± 4.4 |
| Gruvön | 56.3 ± 0.4 | 64.3 ± 0.9 | 68.8 ± 1.0 | 67.2 ± 0.6 | 63.9 ± 0.2 | 64.1 ± 4.3 |
| Gävle | 55.8 ± 1.9 | 53.3 ± 0.2 | 59.2 ± 0.2 | 61.8 ± 0.7 | 46.9 ± 1.1 | 55.4 ± 5.1 |
| Iggesund | 56.3 ± 0.3 | 53.7 ± 1.3 | 57.0 ± 0.7 | 53.2 ± 0.6 | 55.2 ± 0.8 | 55.1 ± 1.5 |
| Karlsborg | 33.5 ± 0.4 | 31.7 ± 0.1 | 32.0 ± 0.5 | 59.9 ± 0.1 | 30.1 ± 0.6 | 37.4 ± 11.3 |
| Munksund | 41.6 ± 0.1 | 36.9 ± 2.3 | 47.5 ± 1.1 | 31.8 ± 0.1 | 35.8 ± 1.5 | 38.7 ± 5.4 |
| Obbola | 38.2 ± 0.1 | 36.4 ± 0.4 | 38.7 ± 0.0 | 33.1 ± 0.2 | 26.8 ± 0.1 | 34.6 ± 4.4 |
| Piteå | No sample | 50.6 ± 0.1 | 47.2 ± 0.2 | 63.9 ± 0.3 | 29.7 ± 0.8 | 47.9 ± 12.2 |
| Skoghall | No sample | 29.1 ± 0.5 | 45.8 ± 0.2 | 47.3 ± 0.3 | 28.5 ±0.5 | 37.7 ± 8.9 |
| Skutskär | 64.7 ± 0.0 | No sample | No sample | No sample | No sample | 64.7 |
| Vallvik | 74.8 ± 0.0 | 63.0 ± 0.4 | 71.4 ± 0.0 | 72.1 ± 0.3 | 64.5 ± 0.1 | 69.2 ± 4.6 |
| Väja | 48.0 ± 13.2 | No sample | No sample | 30.5 ± 0.3 | No sample | 39.3 ± 8.8 |
| Östrand | 33.2 ± 0.1 | 34.0 ± 0.3 | 53.3 ± 0.4 | 35.1 ± 0.2 | 33.7 ± 0.4 | 37.9 ± 7.7 |

Table 2 Minimum, 10^th^ percentile, average, median and 90^th^ percentile and maximum for major elements in solid GLD (Stahre et al., 2024) in mg/kg d.w.

|  | **Al** | **Ca** | **Fe** | **K** | **Mg** | **Mn** | **Na** |
| --- | --- | --- | --- | --- | --- | --- | --- |
| Minimum | 265 | 49 200 | 909 | 498 | 6 570 | 2 400 | 9 050 |
| 10^th^ percentile | 1 690 | 101 500 | 1 050 | 830 | 10 300 | 3 870 | 20 100 |
| Average | 5 070 | 211 000 | 4 060 | 4 790 | 42 800 | 17 900 | 54 600 |
| Median | 4 340 | 216 000 | 3 710 | 3 240 | 32 000 | 17 100 | 45 000 |
| 90^th^ percentile | 9 100 | 341 000 | 7 400 | 9 800 | 95 300 | 36 400 | 106 000 |
| Maximum | 18 200 | 383 000 | 16 400 | 25 800 | 143 000 | 47 100 | 162 000 |
| Samples >DL | 71 | 71 | 71 | 71 | 71 | 71 | 71 |

Table 3 Minimum, 10^th^ percentile, average, median and 90^th^ percentile and maximum for trace elements in solid GLD (Stahre et al., 2024) in mg/kg d.w.

|  | **Ag** | **As** | **Ba** | **Bi** | **Cd** | **Co** | **Cr** | **Cu** | **Ga** | **Mo** | **Ni** | **Pb** | **Rb** | **Se** | **Sr** | **Te** | **Tl** | **U** | **V** | **Zn** |
| --- | --- | --- | --- | --- | --- | --- | --- | --- | --- | --- | --- | --- | --- | --- | --- | --- | --- | --- | --- | --- |
| Minimum | 0.720 | 0.100 | 128 | 0.010 | 1.05 | 1.00 | 17.0 | 30.9 | 0.70 | 0.09 | 7.20 | 2.10 | 2.20 | 0.200 | 169 | 0.010 | 0.040 | 0.050 | 11.0 | 172 |
| 10^th^ percentile | 1.19 | 0.200 | 200 | 0.010 | 2.11 | 2.00 | 33.0 | 45.5 | 1.30 | 0.410 | 9.80 | 3.40 | 5.30 | 0.200 | 222 | 0.010 | 0.109 | 0.109 | 11.8 | 497 |
| Average | 5.88 | 0.300 | 450 | 0.060 | 8.73 | 10.7 | 121 | 233 | 5.51 | 1.00 | 70.6 | 19.4 | 25.6 | 0.200 | 412 | 0.017 | 0.452 | 0.827 | 15.0 | 2 130 |
| Median | 4.86 | 0.300 | 335 | 0.040 | 6.74 | 9.30 | 110 | 202 | 4.80 | 0.740 | 67.1 | 13.5 | 16.7 | 0.200 | 336 | 0.020 | 0.290 | 0.370 | 15.0 | 1 930 |
| 90^th^ percentile | 11.2 | 0.500 | 815 | 0.128 | 17.0 | 19.7 | 237 | 453 | 11 | 1.84 | 131 | 42.6 | 45.6 | 0.200 | 69 | 0.020 | 0.842 | 2.38 | 18.2 | 4 040 |
| Maximum | 22.1 | 0.700 | 1 670 | 0.350 | 32.5 | 40.3 | 418 | 687 | 17.7 | 3.83 | 257 | 92.3 | 163 | 0.200 | 1 240 | 0.040 | 2.42 | 5.04 | 19.0 | 6 030 |
| Samples >DL | 71 | 52 | 71 | 53 | 71 | 71 | 71 | 71 | 71 | 71 | 71 | 71 | 71 | 3 | 71 | 13 | 70 | 60 | 2 | 71 |
